# Pilot study testing the emotional response to physical exercise following a negative emotional induction in adults with borderline personality disorder

**DOI:** 10.1192/j.eurpsy.2021.1170

**Published:** 2021-08-13

**Authors:** S. St-Amour, L. Cailhol, P. Bernard

**Affiliations:** 1 Physical Activity Sciences, Université du Québec à Montréal, Montréal, Canada; 2 Psychiatry, IUSMM, Montreal, Canada

**Keywords:** Borderline personality disorder, Physical Activity, Emotional Regulation

## Abstract

**Introduction:**

Physical exercise is a well-documented treatment for individuals with mental disorder. It helps improve symptoms and functioning of these individuals. Moreover, recent studies indicated that exercise improve emotional regulation which is one of the main target in borderline personality disorder (BPD) treatment. Therefore, exercise might have important benefits in this population. However, no previous study examined this effect.

**Objectives:**

This pilot study documents the facceptability of a protocol testing the effects of exercise on the response to a negative emotion in adults with BPD.

**Methods:**

28 adults with a diagnosis of BPD have been recruited in a psychiatric hospital. Participants filled several questionnaires then viewed a scene from Silence of the Lambs to induce negative emotions. They were then assigned to 20 minutes of exercise or a neutral video of 20 minutes. Affects were assessed 7 times during the protocol.

**Results:**

In this sample, 9 participants reported at least equal levels of affect after the induction than before. Preliminary results show a tendency of higher response of physical exercise than control on positive affects and no participant had any adverse effect from exercise.

**Conclusions:**

This pilot study was the first to test the effects of exercise on symptoms of BPD. It also informs on the best way to conduct the principal study. First, the mood induction was poor, thus it will be changed for a stronger induction strategy. Then, the control intervention will be a placebo exercise. These modifications will enable a better understanding of the effects of exercise on emotion regulation with BPD population.

